# An Efficient Correlation-Based Cache Retrieval Scheme at the Edge for Internet of Things †

**DOI:** 10.3390/s20236846

**Published:** 2020-11-30

**Authors:** Ngoc-Thanh Dinh, Young-Han Kim

**Affiliations:** School of Electronic Engineering, Soongsil University, Sangdo-dong, Dongjak-Gu, Seoul 06978, Korea; younghak@dcn.ssu.ac.kr

**Keywords:** Internet of Things, wireless sensor networks, cache retrieval, information objects, cache reusage

## Abstract

Existing caching mechanisms considers content objects individually without considering the semantic correlation among content objects. We argue that this approach can be inefficient in Internet of Things due to the highly redundant nature of IoT device deployments and the data accuracy tolerance of IoT applications. In many IoT applications, an approximate answer is acceptable. Therefore, a cache of an information object having a high semantic correlation with the requested information object can be used instead of a cache of the exact requested information object. In this case, caching both of the information objects can be inefficient and redundant. This paper proposes a caching retrieval scheme which considers the semantic information correlation of information objects of nodes for cache retrieval. We illustrate the benefits of considering the semantic information correlation in caching by studying IoT data caching at the edge. Our experiments and analysis show that semantic correlated caching can significantly improve the efficiency, cache hit, and reduce the resource consumption of IoT devices.

## 1. Introduction

Information-centric networking (ICN) [1,2] has been considered as a potential networking model for Internet of Things (IoT) applications, not only due to its large namespace for IoT addressing but also its efficient, slim and information-centric networking stack. The majority of IoT traffic comes from the content retrieval, instead of end-to-end communication. In fact, IoT application users who request for content, normally cares about the content itself and how they can retrieve the requested content quickly, not where the content is stored. In ICN, users request for contents based on the name, not the exact location (i.e., like an IP address). The ICN network architecture focuses on the content itself rather than where it is physically located. For that, the ICN core uses the information-centric naming instead of the host-based IP addressing.

The ICN paradigm names content objects and uses their name in routing. In addition, ICN decouples the location of content repository and content publisher from request forwarding. The naming and decoupling features enable ICN to implement in-network caching [3,4,5,6,7,8] at routers for performance enhancement. The in-network caching brings a great benefit of ICN that content objects can be retrieved from any middleware based on the name. By implementing in-network caching, some application requests can be satisfied by cached content objects (COs) at intermediate routers. These requests are forwarded in a shorter distance to get their requested contents and are satisfied faster in comparison with requests that are forwarded to original content publishers. As a result, in-network caching potentially decreases the server load and content retrieval latency which are critical in IoT due to the low-power and lossy network environments. In-network caching is thus considered as the core part of the ICN architecture for IoT.

Some ICN designs propose to cache all COs that forwarded through a router, namely the universal caching policy, so that cached COs are used to serve future interests [1,2]. However, studies have shown that the universal caching policy is inefficient because the caching storage capability of routers, especially in IoT, is normally limited [9,10,11,12,13]. Recently, a number of caching schemes have been proposed to improve the performance of ICN in-network caching and to adapt ICN caching schemes to IoT [2,4,14,15,16,17,18].

Existing studies investigate mostly the two main perspectives of in-network caching, that are caching decision mechanism and cache replacement mechanism [3]. The caching decision mechanism works when a router receives a CO and it has to decide whether to cache the CO or not. Cache Everywhere Everything (CEE) [2] and Probabilistic Caching [3,18] are popular schemes of ICN caching decision. The cache replacement mechanism works when a new CO is reserved to be cached and the cache storage is full. It has to decide which existing cached COs should be replaced with the new one. Random Replacement (RR), Least Recently Used (LRU) and Least Frequently Used (LFU) [3,19] are popular schemes of ICN cache replacement. Another perspective of ICN in-network caching is cache retrieval. A cache retrieval mechanism works when a router receives an Interest message. The cache retrieval mechanism performs cache matching between the Interest message and its CS items. It has to decide whether cached COs in the router’s CS can serve the Interest or not. Existing ICN caching schemes [3,6] use the default exact matching technique to find a cache for an Interest. Following the technique, a cache hit takes place at a router only when the requested content and one of its cached COs are matched exactly. This is due to the conventional Internet content consumer model where consumers request for a specific content object. We find that this usage does not translate well to the IoT.

Previous studies have shown that in many practical IoT scenarios (i.e., building management, mobile computing, pervasive computing applications, …), applications and users who are interested in sensing content, normally allow a level of the error tolerance for data accuracy [20,21]. It means that a given data accuracy requirement of an application can be specified. As a result, in IoT, a cached content object which has a high correlation with the requested content and meets the application requirement, can be reused for a cache retrieval. A cached content object of a sensor is reusable for similar application requests to other sensors of the same type if the cached content object satisfies the data accuracy (DA) requirements of the applications. This is beneficial in IoT because sensing data of sensors of the same type normally have a high correlation (i.e., temperature sensors deployed in a building).

This paper proposes an efficient correlation-based cache retrieval (CCR) scheme which considers the semantic information correlation of information objects of nodes for cache retrieval. We develop a completed cache retrieval mechanism based on the preliminary idea [22]. The detailed procedures of cache retrieval, application model, ICC discovery, new prototype implementation with new evaluation results are presented. By exploiting the semantic information correlation, we extend the concept of cache hit. For a given content request from an application, a cache hit occurs at a node if it has a cached content object that meets the application request requirement, not necessarily the exact object that the application requests. The proposed cache retrieval mechanism enables the re-usability of available cached items to serve more diverse requests based on information correlation. We illustrate the benefits of considering the semantic information correlation in cache retrieval by studying IoT data caching at the edge. Our obtained experimental results show that the proposed cache retrieval mechanism can significantly improve the efficiency, cache hit, and reduce the resource consumption in IoT.

## 2. Related Work

Information-centric networking (ICN) has been demonstrated benefits for IoT applications, thanks to its name-based forwarding, slim network stack, and especially in-network caching. Apart from reducing the network load and increasing content redundancy, in-network caching helps lower data delivery delay by making cached content objects readily available throughout the network [23]. In-work caching operates under the fact that a content is normally useful for a time period and is requested by multiple consumers during its lifetime. One of interesting question in in-network caching is where content objects should be cached. In the literature, many studies have investigated caching schemes for the edge and the core. Studies [9,24,25,26] shows that the edge is one of the best places for storing content objects. The reason is that caching content objects closer to consumers makes more sense. If contents are stored at the producer or nearby the producer, the traffic load to the producer will be high and the data delivery latency will be long. If there are consumers requesting a content from a specific region in the network, consumers in the region normally have demand for the content again in the future. Therefore, if the content is cached at the edge of the region, requests for the content will be retrieved faster.

A caching strategy consists of three processes: caching decision, cache replacement decision, and cache retrieval. The caching decision is made once a node receives a CO. The node has to decide to cache the CO in its content store or discarded. Cache Everything Everywhere (CEE) and Probabilistic Caching (ProbCache) are two popular caching decision mechanisms in ICN. The idea behind CEE is very simple. Following CEE, an ICN node tries to cache every new CO it receives, that has not cached in its CS. ProbCache introduces a certain probability of caching for a CO it receives. For a given priori probability p, When an ICN node receives a new CO, it randomly generates a number between 0 and 1. If the value is smaller than p, the node makes a decision to cache the CO. Otherwise, the node discards the CO. An important implicit indication is that ProbCache should give a higher priority of caching to more popular contents which are requested more often.

The cache replacement decision is made once a CO is decided to be cached, but the CS is full. Then the node has to decide which existing cached items should be deleted and replaced by the new CO. LRU (Least Recently Used) and LFU (Least Frequently Used) are two popular cache replacement mechanisms in ICN. In LRU, a node keeps track of Interest requests it forwards and the time it receives. LRU then makes a cache replacement on a cached item that was least requested recently. This means that unpopular cached items should be evicted for more fresh and popular COs to be cached. In LFU, a node monitors the frequency of requests for cached items. When a cache replacement is required, LFU deletes the least frequently used one. There are several less popular cache replacement mechanisms like RR (Random Replacement) and MDMR (Max Diversity Most Recent). In RR, a node simply removes a random cached item to have space to cache a new CO. In MDMR, a node tries to delete an older cached item from the same publisher or simply delete the oldest cached item.

The cache retrieval performs cache matching for the requested content between the Interest message and contents stored in the content store of a node. The cache matching determines whether a cache can be used to satisfy a request. Existing caching mechanisms utilize exact cache matching because, in the conventional content consumer model, consumers request for specific contents. However, we find there are some use cases where correlated contents can be used and thus propose a semantic correlation-based cache retrieval mechanism for IoT.

Detailed literature reviews of this topic can be found in [3,4,6,7].

## 3. The Proposed Correlation-Based Cache Retrieval

In this section, we describe the design of the proposed correlation-based cache retrieval (CCR) scheme. We first conduct a testbed experiment and statistic to illustrate the sensing data correlation in real-world deployments. We then discuss the application model of CCR. CCR is implemented on the top of CCN. The processes of CCR are as follows. CCR first discovers information correlated communities (ICCs) [20] among sensors based on the interest and data accuracy (DA) requirements of applications. The ICC discovery process is presented in the Section 3.4. CCR then constructs an ICC caching table which stores a list of ICCs and their corresponding cache pointers. The detail of the ICC caching table is described in the Section 3.5. An ICC-based cache retrieval mechanism is then presented in the Section 3.6, which extends the current cache hit concept to enable an application request sent to a member of an ICC be able to take advantages of any available cached content object (CO) of the ICC. The list of acronyms is presented in Table 1.

### 3.1. An Illustration for Sensing Data Correlation

We conduct testbed experiments to study the correlation of sensor data in real-world scenarios. For this illustration, we deploy a sink node and 40 TelosB nodes including temperature and humidity sensors around a building. Nodes are labeled with the ID from 1 to 40. Sensors are configured to run CTP [27] for sensing data collection and deployed randomly at different positions around the building indoor space as described in our previous study [28]. In each deployment, sensing data are collected and analyzed for two hours. After multiple experiments, we observe that high sensing data correlation is quite popular among sensors deployed in similar spaces (i.e., in an indoor space). For examples, a number of temperature sensors deployed in different rooms in the same floor of the building show quite similar sensing data over the time. Most of temperature and humidity sensors in the experiments show that their sensing data have a high correlation with sensing data of several other sensors of the same type. However, the correlation degrees of groups of sensors are different. For example, a group of temperature sensors shows a correlation degree of over 95% while another group shows a correlation degree of only 85%. We also observe that some sensors may have a low sensing data correlation with other nodes.

Table 2 illustrates the sensing data correlation of various groups of sensors observed in our experiments. In Table 2, the first, second, and fourth group show a very high sensing data correlation of over 95%. The third and fifth group show a slightly lower sensing data correlation of over 85%. The sixth group of the 17-th sensor shows a low sensing data correlation compared to other nodes: 34-th, 38-th, and 39-th. This is due to the fact that the 17-th sensor is deployed nearby a humidifier, so its humidity value is much higher than the humidity value of other humidity sensors in the same floor.

### 3.2. Application Model

In IoT scenarios, WSNs are deployed to provide sensing information to applications. A WSN can be deployed to provide sensing information for multiple applications at the same time. According to previous studies, the edge architecture is an appropriate one for IoT where the edge node is deployed to serve as a middleware connecting applications with one or several WSNs. State-of-the-art studies [3,4] suggested that the edge node is one of the best options to serve as a caching node for IoT data.

Assume we have a set *N* of n application requests. Each request is sent to request for a set of sensing data types st provided by a WSN. The applications may have different requirements of the Data Accuracy (DA) level. For example, some applications request for a DA level of 95% while other applications may accept a DA level of 90%. Note that the price of sensing services is usually proportional to the data quality requirement of applications based on a service level agreement (SLA) [20,29]. For example, the higher the data accuracy level is requested, the higher the price is charged. In summary, the set of application requests is denoted as $N=\left(a_{1},st_{1},DA_{1}\right),\left(a_{2},st_{2},DA_{2}\right),...,\left(a_{n},st_{n},DA_{n}\right)$.

The proposed scheme performs aggregation of all application requests to find a consolidated DA cDA requirement (i.e., the greatest DA requirement among application requests) which satisfies all application requirements. Therefore, by grouping ICCs based on the consolidated DA requirement (cDA), data of a member of an ICC can satisfy requirements of all applications requesting sensing data from one of the ICC’s members. Once the scheme finds a new cDA, the cDA value is used as the input parameter for ICC discoveries. The detailed operations of the application model and aggregation scheme can be found in our previous studies [20], so we don’t repeat the detail in this paper.

### 3.3. Node Naming and Content Object Naming

We utilize the naming syntax in [22,30] to name content objects and sensors. The name structure for a node consists of two elements, the category prefix (CP) and the node ID. The category prefix indicates the real-world category (i.e., temperature sensor or humidity sensor) of the node. The node ID makes the name of the node unique and persistent. We specify the brief category prefixes for sensor types, for example, “temp” for temperature sensors, “humi” for humidity sensors, “ligh” for light sensors,... We assume that a standardized list of sensor types is available. For instant, the name of a temperature sensor can be “Temp::1325”. Note that following the above structure, both flat and hierarchical style can be use to name a sensor node. In this paper, we use the flat style for naming.

We associate the name of content objects (CO) of a sensor with its name. The name of a sensor is also used to name its content objects (CO). This is due to the fact that a sensor produces a type of content over time. Each time the sensor executes sensing and produces a new version of the content. As a result, we use an additional sequence number for the CO to indicate the version of the CO. In our implementation, a CO of the temperature sensor “temp::1325” has the name with a sequence number as “temp::1325/*p* = 0087”. The sequence number is increased by the time. A new content has a greater sequence number than its old version. The sequence number is designed to enable consumers and content providers to know the latest version of content produced by a node.

### 3.4. ICC Discovery

We first define the concept of an ICC as follows. The ICC of a node *k* (i.e., $ICC_{k}^{r}$) is defined as a set of nodes *h* that have sensing information correlation greater than or equal to *r* compared to the sensing information of *k*. Therefore, the sensing data of *k* can be exploited for serving application requests with the DA requirement lower than or equal to *r* for nodes in $ICC_{k}^{r}$. Based on their correlation, data of a node *j* in $ICC_{k}^{r}$ can also be used in the reconstruction of the data of node *k*. The ICC discovery was presented in detail in our previous study [20,31,32]. We briefly describe our implementation approach for the ICC discovery below.

In our implementation, we limit the ICC discovery of a node *k* within the sensor type community (STC) of node *k* to reduce the overhead. An STC of a node *k* is defined as a group of nodes with the same sensor type with *k* (i.e., temperature sensor). The sensor types are predefined. The STC discovery can be implemented easily using the naming scheme described in our previous work [30] in which nodes with the same sensor type (i.e., temperature) have the same category prefix (i.e., “temp:*”). Nodes with the same category prefix are grouped into the same STC.

Our ICC discovery is based on the network community discovery theory [20,31,32,33]. Given a set of *N* nodes N=1,2,...,n, a node k(k∈N), and its data points $k_{i}$, we determine a regression function ω and a community of node k, ICC $ICC_{k}$, such that the expected value of the loss function $\Phi \left(d,\omega \right)=Loss(d_{k},\omega \left(d_{STC_{k}}\right))$, is minimized.

We assume that ω is linear and Φ is mean square error (MSE). We then find a set of nodes $ICC_{k}\in N\backslash k$ so that there is a decision ζ minimizing.

(1) $$E\left[\Phi \left(D,\zeta \right)\right]=E\left[{\left(D_{k}-\zeta^{T}D_{ICC_{k}}\right)}^{2}\right]$$

In the equation, $D_{X}$ is a random vector consisted of ${\left\{D_{k}\right\}}_{k\in X}$. The ICC discovery process is executed based on a historical sensing data set with m samples $D=[d_{1}d_{2}...d_{m}]$. A heuristic solution is implemented to find the decision $\zeta_{k}$ based on the data set so that it can find the number of zero entries of ζ as great as possible. In other words, we should determine a sparse decision $\zeta_{k}$ to minimize the MSE and the $L_{1}$ norm, which is a typical Least Absolute Shrinkage and Selection Operator (LASSO) problem [34]. Therefore, the problem can be solved easily by introducing a LASSO parameter ρ as follows.

(2) $$minimize\{\rho \|\zeta_{k}{\|}_{1}+1/2\|d_{\left[-k\right]}\zeta_{k}-d_{k}{\|}_{2}^{2}\}$$

As a result, the $ICC_{k}$ is determined as a group of nodes with non-zero entries of $\zeta_{k}$. The parameter ρ is used to control the expected error (i.e., the data error tolerance or DA requirement) as well as the sparsity of ζ that affects the size of $ICC_{k}$.

### 3.5. ICC Caching Table

Based on the set of application requests, $N=\left(a_{1},st_{1},DA_{1}\right),\left(a_{2},st_{2},DA_{2}\right),...,\left(a_{n},st_{n},DA_{n}\right)$, the edge performs the ICC discovery for sensor types and their corresponding data accuracy requirements, using the ICC discovery procedures described above. The edge then constructs an ICC caching table as shown in Table 3.

The ICC caching table consists of a list of ICCs which are discovered based on the sensing type and the consolidated DA requirement of corresponding applications. The consolidated DA requirement thus represents the content correlation among nodes in an ICC. Each ICC in an entry of the table has a cache pointer which points to the corresponding cached CO if one of the members of the ICC has cached COs. If there is no member of the ICC has cached COs, the cache pointer is NULL. Note that the ICC caching table can be updated during the run time. As the name of a CO doesn’t change, the edge can update the cache with a new version of the CO by the time (i.e., the CO with a new sequence number) to ensure the up-to-date data. To increase the cache diversity and cache efficiency, only COs of one member within an ICC need to be cached.

### 3.6. Cache Retrieval and Cache Hit

CCR is proposed to evaluate, construct, and enable the re-usability of available cached items in the system to serve more diverse requests based on information correlation in sensing data of sensors. The objectives of CCR are to improve the cache hit at the edge and reduce the number of application requests sent to physical WSNs by exploiting available cached items to serve eligible application requests that the available cached items can satisfy the requests. For that, CCR constructs and uses the ICC caching table for its operations.

**Figure 1:**
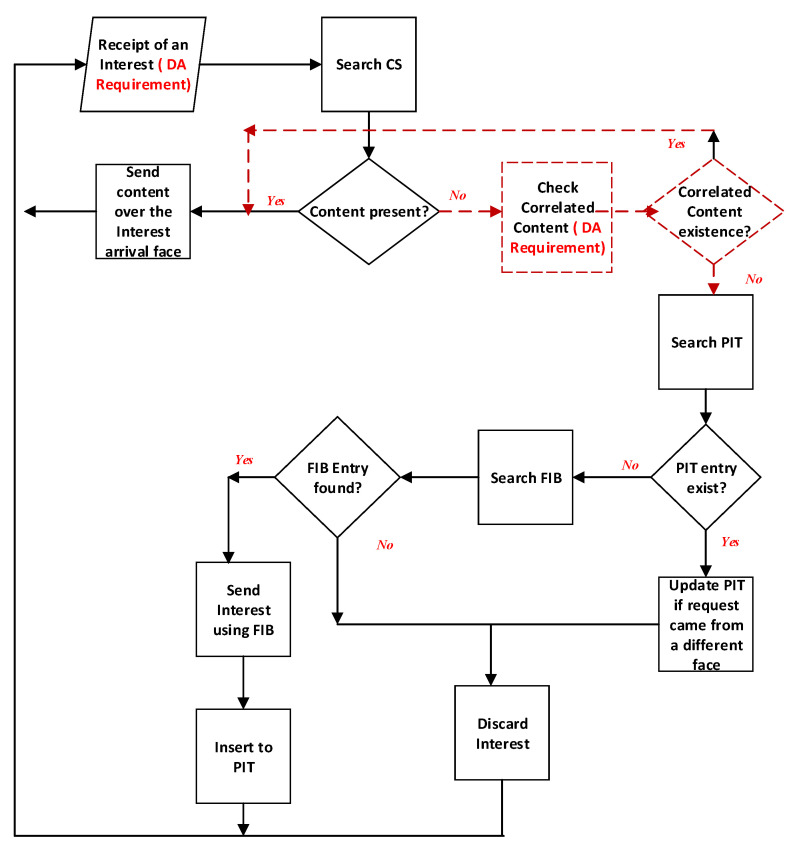
The interest packet processing flow in CCR. In the figure, the dotted red line shows new parts of implementation in comparison with the existing CCN.

The detailed procedures for Interest packet processing of the proposed scheme are presented in Figure 1. When the edge receives an application interest request with a CO name *c* and a DA requirement, the edge checks its content store (CS) for cache retrieval. If the CO is cached in the CS, the edge returns to the request with the cached CO. If there is no cache for the CO name, the edge further explores the ICC caching table. In particular, the edge checks whether or not there is any list containing *c* with the correlation which is greater than or equal to the DA requirement. If there exists a list with a satisfied requirement and has a cache pointer. The edge returns to the request with the cached CO indicated by the cache pointer that satisfies the requirement of applications. If there is no list satisfies or there is no cache, the request is forwarded further based on FIB to the node following the regular operations of CCN/NDN [1,2,35].

In this way, we extend the definition of a cache hit in ICN. For a given content request from an application, a cache hit occurs at a node if it has a cached content object that meets the application request requirement, not necessarily the exact object that the application requests. If a cached content object of the same sensor type has a high correlation with the requested content object, that meets the application requirement, a cache hit occurs. The cached item is then reused to respond to the request.

## 4. Performance Evaluation

We implement a prototype of CCR in Contiki CCN [35] on the top of ProbCache with LRU (Least-Recently-Used) [3,18] and LCD (Leave Copy Down) [4] with LRU. We then perform simulations using COOJA simulator [35] with an edge node and 1500 sensors of temperature, humidity, and accelerometer types. Sensors are deployed randomly with sensing correlation obtained from the sensor data collected from the real-world IntelLab deployment [36] for a natural correlation. The system generates 3000 content requests following Zipf-like distribution with random data accuracy requirements from 80% to 100%. For the ProbCache, we set the caching probability p=0.5. The CS storage capacity is varied from 20 to 180 content objects.

We use the following metrics for the performance evaluation and comparison.

**Cache hit ratio**: measures the percentage of content requests that are satisfied by cached COs in the CS of ICN routers. Cache hit ratio is utilized popularly in the performance evaluation of a caching scheme. A high cache hit ratio indicates a good performance of a cache scheme.

**Server hit ratio**: measures the percentage of content requests that are forwarded to content providers (i.e., sensors) in WSNs. It indicates the number of requests that content providers must generate sensing content in response directly to the requests. At absence of in-network caching, the number of server hits is equal to aggregated content requests from all applications.

**Content retrieval latency**: measures the average time required for content requests to get satisfied whether from a cache or from an original content publisher.

Due to resource constraints, we perform experiments with light-weight sensing data and limited cache capacity. Experiments can be conducted similarly for other types of sensing data like images or videos. As implemented in our prior study [28], HTTP-CoAP converter are reused in this paper for converting HTTP application requests to CoAP requests for sensor nodes. We assume that each application or user demands one of the sensing data types above. We encode application requests using XML templates and decode with SensorML interpreter for sensors [20] For the WSNs, we use CTP and LPL [28] as the sensing data collection protocol and the duty-cycled MAC mechanism. For the radio noise model, we use the closest-fit-pattern matching (CPM) [28]. We set the CCA check parameter up to 400 times. The detailed configurations for simulations are presented in Table 4. Other parameters are kept the same as the default configurations of Contiki CC2420 radio model [28]. The naming scheme [30] is used for sensors to facilitate the ICC grouping. The main modification of our cache retrieval scheme implementation compared to the existing CCN implementation is summarized in Figure 1. The obtained results are reported at 95% confidence interval.

**Figure 2:**
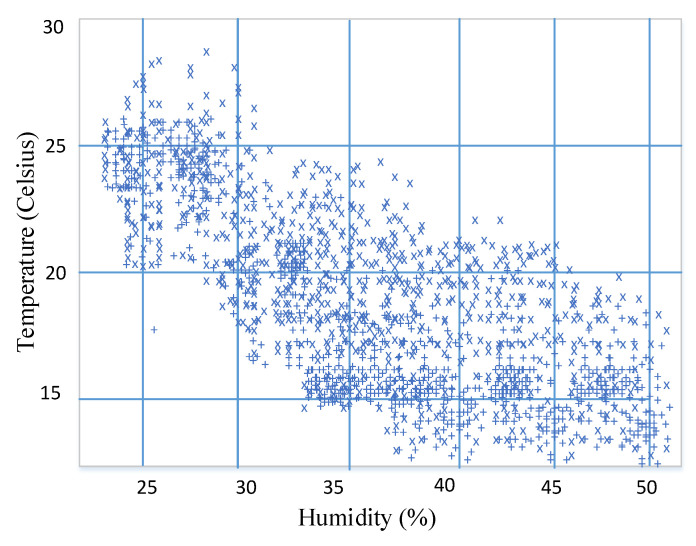
Scatterplots of temperature and humidity sensing data.

Figure 2 shows the distribution of the temperature and humidity sensing data in the experiments.

### 4.1. Cache Hit Ratio

**Figure 3:**
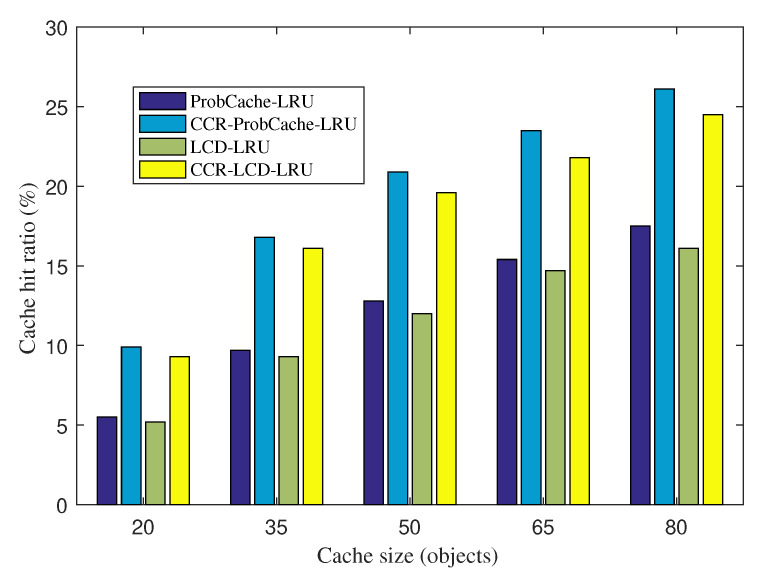
Cache hit ratio vs. Cache size.

Figure 3 shows the average cache hit ratios of ProbCache-LRU and its version with CCR on the top, CCR-ProbCache-LRU, and LCD-LRU with CCR-LCD-LRU under various cache sizes. Obtained results show that CCR helps the system improve the cache hit ratio significantly within a limited cache capacity. For all cache sizes, the cache hit ratios witnessed with CCR-ProbCache-LRU and CCR-LCD-LRU are higher than ProbCache-LRU without CCR and LCD-LRU without CCR. Note that all cache hits in CCR-ProbCache-LRU, ProbCache-LRU, CCR-LCD-LRU, and LCD-LRU satisfy the applications’ requirements. The performance improvement by CCR-ProbCache-LRU and CCR-LCD-LRU in comparison with ProbCache-LRU and LCD-LRU is achieved due to the following reasons. CCR takes advantages from the data accuracy tolerance of IoT applications. Based on that, CCR explores content producers having a high content correlation to establish information correlated communities (ICCs). CCR then establishes an ICC caching table to enable the edge to exploit a CO for serving application requests that it can satisfy. In this way, a cached item in CCR can serve not only application requests with the exact name of interest but also similar requests of the same type with a satisfactory data accuracy requirement.

When we increase the cache size, the cache hit ratios of CCR-ProbCache-LRU, ProbCache-LRU, CCR-LCD-LRU, and LCD-LRU increase gradually. This phenomenon is easy to understand because with a higher cache size, the CS can store more diversified content objects and can satisfy various application requests. An interesting point is that the cache hit ratio improvement of CCR-ProbCache-LRU over ProbCache-LRU and CCR-LCD-LRU over LCD-LRU decreases when we increase the cache size. In particular, CCR-ProbCache-LRU achieves the cache hit ratio improvement of 79.2% at the cache size of 20 COs. The improvement decreases to 73.1%, 62.8%, 54.3%, and 47.6% when the cache size is increased to 35, 50, 65, and 80 COs, respectively. The similar trend is observed with CCR-LCD-LRU and LCD-LRU. This indicates that CCR-ProbCache-LRU and CCR-LCD-LRU achieve a higher improvement percentage in cases with a limited cache size. It is easy to predict that when the cache size is increased equal to the content population, the improvement percentage is decreased to 0. The phenomenon is reasonable. The reason is that when all COs can be cached, exploiting correlated content is unnecessary and there is no benefit of ICC caching table. However, in real scenarios, network nodes normally have a limited cache size in comparison with the content population.

### 4.2. Server Hit Reduction Improvement

**Figure 4:**
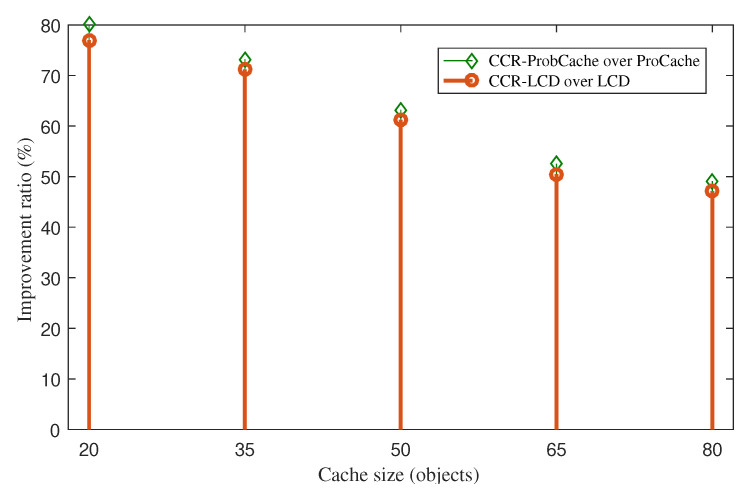
Server hit reduction improvement of CCR-ProbCache-LRU over ProbCache-LRU.

The results of the cache hit ratio can also be translated into the server cache hit ratio. The higher the cache hit ratio leads to the lower the server hit ratio. As a result, CCR also helps in reducing a significant number of application requests sent to resource-constrained sensors. The improvement ratios of server hit reduction of CCR-ProbCache-LRU over ProbCache-LRU and CCR-LCD-LRU over LCD-LRU are presented in Figure 4. The figure shows that the systems with CCR witness a considerable smaller number of application requests sent to sensor nodes. The improvement ratios are maintained over 40% corresponding with the cache size from 20 to 80 content objects. When the cache size increases, the impact of CCR decreases. It does mean that the lower the cache capacity the higher the improvement ratio CCR achieves. The server hit reduction improvement reflects that CCR saves a significant amount of energy consumption of sensors to process direct requests that are sent to them.

### 4.3. Content Delivery Latency

**Figure 5:**
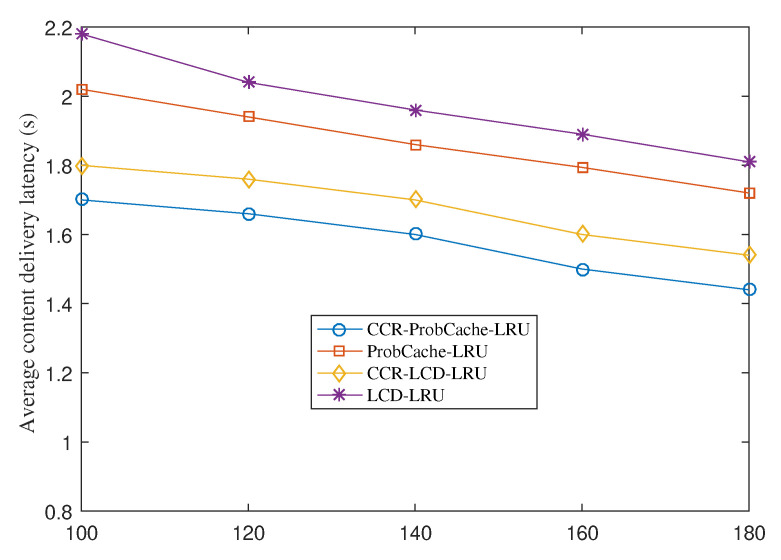
Average content delivery latency vs cache size.

Figure 5 presents the average content delivery latency under various cache sizes of CCR-ProbCache-LRU, ProbCache-LRU, CCR-LCD-LRU, and LCD-LRU. When the cache size increases, the content delivery latency in ProbCache-LRU, CCR-ProbCache-LRU, LCD-LRU, and CCR-LCD-LRU decreases gradually. The reason is that the higher the cache size the greater the number of COs the systems can store. This results in a greater number of application requests are satisfied by cached COs. The cache retrieval of cached COs consumes a short time period because the content is retrieved at the edge immediately instead of forwarding requests to sensors. By increasing the cache hit ratio at the edge, the systems with CCR respond to application requests faster than that without CCR. CCR-ProbCache-LRU and CCR-LCD-LRU achieve a significant improvement in term of average content delivery latency in comparison with ProbCache-LRU and LCD-LRU, respectively.

## 5. Discussion and Conclusions

This paper considers the semantic correlation between content objects in IoT, which is popular, for cache retrieval and cache re-usability. Based on the semantic correlation between content objects, we extend the concept of cache hit to improve the performance and energy efficiency of IoT devices. In the proposed cache retrieval mechanism, a cached content object can be reused to serve more diverse requests as long as the content object satisfies the requirement of application requests. The obtained results show the potentiality of considering the semantic correlation between content objects in caching in IoT. In the future works, we plan to use cross layer approaches to explore the benefits of considering the semantic correlation between content objects at the lower layer and upper layers. We then move toward building a completed semantic protocol stack for WSNs based on the semantic content correlation and design a simple way to build applications on the top of the stack.

## Figures and Tables

**Table 1:** List of Acronyms.

Acronym	Meaning
ICN	information-centric networking
CO	content object
DA	data accuracy
iIPV	the IPV of an inactive IPD
ICC	information correlated communities
CTP	collection tree protocol
CP	category prefix
STC	sensor type community
CCR	correlation-based cache retrieval
CS	content store
FIB	forwarding information base
PIT	pending interest table

**Table 2:** An illustration for sensing data correlation in an indoor testbed experiment.

Sensor i	Correlated Nodes (have the Max Correlation with i)	Type	Correlation Degree
1	2, 8, 26, 32	Temperature	95%
3	7, 11, 16	Humidity	95%
4	2, 6, 10, 11, 16, 27	Temperature	87%
5	9, 14, 32	Temperature	95%
12	15, 24, 36	Humidity	85%
17	34, 38, 39	Humidity	64%

**Table 3:** Illustration of an ICC caching table (“A*” indicates the pointer to the cached content of A).

Prefix	Correlation	ICC Lists	Cache Pointer
Temp	97%	ICC1 (ID2, ID5, ID8)	ID2 *
Temp	97%	ICC2 (ID1, ID3, ID9, ID12, D15)	ID3 *
Temp	97%	ICC3(ID4, ID6, ID13)	ID4 *
Ligh	94%	ICC4 (ID7, ID16, ID18)	ID7 *
Ligh	94%	ICC5 (ID10, ID21, ID24, ID25)	NULL
Humi	96%	ICC6(ID22, ID29, ID32)	ID29 *

**Table 4:** Parameters.

Parameter	Value	Parameter	Value
$DA_{c}^{apps}$	85–100%	Cachesize	20–200 objects
channel sampling	10 ms	ProbCache p	0.5
$T_{w}$	2 s	α	0.95
simulation time	6 h	correlation	0.8–1
